# Religion Welcome Here: A Pluriversal Approach to Religion and Global Bioethics

**DOI:** 10.1007/s11673-024-10410-7

**Published:** 2024-12-16

**Authors:** N. S. Jecker, C. A. Atuire, V. Ravitsky, M. Ghaly, V. Vaswani, T. C. Voo

**Affiliations:** 1 African Centre for Epistemology and Philosophy of Science, University of Johannesburg, Johannesburg, South Africa; 2 Centre for Bioethics, Chinese University of Hong Kong, Hong Kong, China; 3 Department of Bioethics and Humanities, University of Washington School of Medicine, 1959 NE Pacific Street, Campus Mailstop 357120, Seattle, Washington, 91895-7120 USA; 4 Department of Philosophy and Classics, University of Ghana, Accra, Ghana; 5 Centre for Tropical Medicine and Global Health, University of Oxford, Oxford, UK; 6 Department of Global Health and Social Medicine, Harvard Medical School, Boston, MA USA; 7 The Hastings Center, Garrison, NY USA; 8 Research Center for Islamic Legislation & Ethics, Hamad Bin Khalifa University, Ar-Rayyan, Qatar; 9 Centre for Ethics and Department of Forensic Medicine and Toxicology, Yenepoya University, Mangaluru, India; 10 Office of Ethics in Healthcare, SingHealth, Singapore, Singapore

**Keywords:** Pluriversal view, Religion, Global bioethics, Epistemic justice, Public reason

## Abstract

This paper sets forth and defends a pluriversal approach to religion in the context of an increasingly global bioethics. Section I introduces a pluriversal view as a normative technique for engaging across difference. A normative pluriversal approach sets five constraints: civility, change from within, justice, non-domination, and tolerance. Section II applies a pluriversal approach to religion. It argues that this approach is epistemically just, recognizes diverse standpoints, and represents a productive, preferred, way to tackle global bioethics concerns. Section II also considers an opposing viewpoint, which holds that religious perspectives have no place in bioethics. We show that this viewpoint would have adverse effects on bioethics publishing, conferencing, and training programmes. The paper concludes (in Section III) that bioethicists should engage with people who hold different worldviews, including religious worldviews, and should do so in accordance with pluriversal ethical constraints.

## Introduction

Religious scholars have figured prominently in bioethics since its inception. During the mid-twentieth century, often considered bioethics’ “birth,” Jonsen describes a “trinity of theologians” presiding over bioethics creation: “Joseph Fletcher, an Episcopal minister; Paul Ramsey a Methodist professor; and Richard McCormick, a Jesuit moral theologian” (Jonsen 2003, 41). While individuals who are religious may engage in bioethics without invoking religious arguments (as Fletcher, for example, did when propounding “situation ethics” (Fletcher 1997)), the fact that these early bioethicists were actively religious suggests perhaps that they were not opposed to or hostile toward religious contributions to bioethics. Today, bioethics is well established within academia and spans multiple disciplines. Yet, over time, the field has increasingly turned away from religious views to methods of law, philosophy, and empirical sciences, and religion’s role has receded (Stempsey 2011; Callahan 1999). The legitimacy of religious contributions to bioethics has recently been questioned and debated (Audi and Smith 2023; Crisp 2023; Savulescu 2023; Schüklenk 2018; Murphy 2012; Crane and Putney 2012; Chapman 2012; Jones and Whittaker 2012; Camosy 2012; Durante 2012; Claasen-Lütner 2012; Cahill 1990). Yet, with rare exceptions (ten Have 2016; Biggar 2015), the literature has not discussed religion’s role in bioethics from a *global* standpoint.

As bioethicists from diverse regions (the United States, Ghana, Qatar, Singapore, Israel, and India), we, the authors, regard religion as a source of morality for many people around the world and thus, key to our field’s ability to gain global traction and address problems that are increasingly global in scope. We elaborate and support this position in Section I by introducing a pluriversal approach. A pluriversal approach invites people with different ways of knowing, being, and acting into bioethics conversations and supports the existence of plural worldviews, including religious worldviews, provided they do not harm people or destroy other worlds. Its methods of engaging imply ethical constraints of civility, change from within, justice, non-domination, and tolerance. Section II applies a pluriversal approach to religion. We argue this approach is epistemically just, recognizes diverse standpoints, and represents a productive, preferred, way to tackle global bioethics concerns. We also argue that an opposing viewpoint, i.e., that religious perspectives have no place in bioethics, would have adverse effects, especially on bioethics publishing, conferencing, and training programmes. The paper concludes (in Section III) that bioethicists should engage with people who hold diverse ways of knowing, being, and acting, including religious ways, and should do so in accordance with pluriversal ethical constraints.

## A Pluriversal Proposal

This section proposes a pluriversal approach to global bioethics. To motivate this approach, consider first, how appealing only to secular reasons to justify an ethical stance may fall short. Suppose you offer me reasons derived from your comprehensive worldview and suppose that worldview is religious. Refusing to consider your reasons because they are religious, would be profoundly disrespectful. Wolterstorff reckons that such a response cannot be right:

Is there not something about the person who embraces, say, the Jewish religion, that I, a Christian, should honor? Should I not honor her not only as someone who is free and equal, but as someone who embraces the Jewish religion? Is she not worth honoring not only in her similarity to me, as free and equal, but in her particular difference from me—in her embrace of Judaism? (Wolterstorff 1997, 110–111)

Honouring persons in their various particularities—whether religious, gender, class, race/ethnicity, or other dimensions of identity, is crucial for respecting them as the actual persons they are and experience themselves to be. Dworkin puts the point thus:

… it is hardly plain that it would be desirable for people of religion to keep their convictions divorced from their politics even if that were possible. Martin Luther King Jr. was a man of faith, and he invoked his religion to condemn prejudice with great effect; Catholic priests speaking as priests have been vanguard fighters for social justice in Latin America. (Dworkin 2008a, b, 65)

Dworkin concludes that asking people of faith to leave their religious convictions behind when they take up their role as citizens would be misguided, because citizenship requires “sincerity and authenticity, which is impossible for [religious] people unless they keep their religion very much in mind” (Dworkin 2008a, b, 65).Analogously, inviting religious people to engage in *bioethics* while leaving their religion behind would be misguided because some regard being a bioethicist as *inseparable* from being a religious person.

Wolterstorff and Dworkin’s remarks capture important insights. First, for some religious people, religion is not exclusively about a private interior faith; it is also about ethical commitments that have impact in the public square. Second, social identities, which may include religious dimensions, can be constitutive of who a person is and experiences themselves to be. These insights suggest the need to re-examine the almost reflexive tendency among some bioethicists to exclude religion.

Building on recent work in Latin American decolonial studies and critical anthropology, we propose that religious and secular approaches reflect not just different worldviews but different ontologies or existences. Pluriversal views generally assert that people live distinct, internally coherent existences, and that these different worlds should coexist, and even flourish, provided they avoid harming people or destroying other worlds.^1^ The underlying idea reflects a commitment to *ontological justice*: sustaining the possibility of plural worlds enacted through language, belief, and knowledge. Escobar explains it thus: “the world is made up of multiple worlds, multiple ontologies or reals that are far from being exhausted by the Eurocentric experience or reducible to its terms” (Escobar 2020, 69).

The point of focusing on ontology, as the concept of “pluriverse” does, is to take seriously the depth of difference. By positing ontologies, however, our concern is less with making metaphysical claims than with asserting the *normative* claim that people inhabiting distinct worlds merit equal recognition and respect. A pluriversal normative approach can be shared by those holding divergent views about what exists and what counts as evidence. It is an approach a scientist could accept because it does not imply endorsing beliefs or methods that a science-based worldview rejects. Nor does a normative pluriversal account assert that all knowledge claims are equally valid, or that “anything goes.” It is not a commitment to epistemological or moral relativism. Instead, a normative pluriversal view focuses on how to ethically engage with people who abide by different ways of knowing, being, and acting. Pluriversality challenges each of us to be ethical, not just within our own world but within a world of many worlds.

To accomplish these aims, a pluriversalist commits to certain ethical constraints for engaging across difference. These constraints support the coexistence of plural worlds provided they do not harm people or destroy other worlds. Some ways of knowing, being, and acting clearly transgress ethical constraints pluriversality sets. For example, Nazi morality, understood as a set of beliefs centred on principles of antisemitism and racial purity, violates the rights and dignity of persons and destroys other worlds. By contrast, consider Blaser’s description of two groups of people who inhabit radically different worlds, yet can coexist within the ethical boundaries of pluriversalism.

In June 2004, in the province of British Columbia, Canada, the Mowachat/Muchalaht First Nation botched a carefully staged and scientifically approved plan by Canada’s Department of Fisheries and Oceans and environmentalist groups to return a young lost orca whale, Luna, to its pack. The First Nation insisted that the orca was Tsux’iit, the abode of the spirit of their recently deceased chief, Ambrose Maquinna, and that his desire to stay with his people should be respected. This was not a conflict between two different perspectives on an animal but rather a conflict over whether the “animal” of scientists… was all that was there (Blaser 2013, 548).

In Blaser’s example (unlike the Nazi example), the Mowachat/Muchalaht First Nation people and the scientists at Canada’s Department of Fisheries can find ways to dialogue respectfully despite deep-seated differences. By framing the conflict between them as a conflict of *worlds,* a pluriversal view takes seriously that Tsux’iit is just as “real” for the Mowachat/Muchalaht First Nation people as Luna is for the scientists at Canada’s Department of Fisheries.

When taking a pluriversal approach to global bioethics, a key task becomes facilitating dialogue across deeply different ways of being, knowing, and acting. Respect and recognition call for judicious judgment: we do not expect First Nation people to leave their spiritual beliefs behind when they discuss Tsux’iit’s fate, nor do we expect people at the Department of Fisheries and Oceans to leave their scientific beliefs behind when they discuss Luna’s fate. Instead, the following five ethical constraints ensure mutual respect and enable radically different worlds to coexist and even flourish (Table 1, and discussion following).

**Table 1:** Ethical constraints a pluriversal approach sets

Ethical Constraint	Definition	Elaboration/Illustration
1. Be civil	Engage others with respect	Show hospitality across a global bioethics community by being welcoming, friendly, open, generous, and kind
2. Identify and support drivers of change from within	Resolve conflict and reduce harm by working with those experiencing them; recognize people’s agency to determine for themselves the best way forward	Respond to the maltreatment of people accused of witchcraft by working with community leaders to bring about reconciliation, and making peace within and between families
3. Be just	Give each their due (dueness condition); act to ensure each feels they are given their due (subjective condition)	Meet the dueness condition by treating like cases alike; meet the subjective condition by taking steps to ensure people feel they are being heard and treated fairly
4. Be non-dominating	Avoid exerting an arbitrary or controlling influence on others	Avoid both religious intolerance and intolerance of religion
5. Be tolerant	Avoid judging others with undue severity	Set forth one’s positions non-dogmatically and be open to revising them if new arguments or evidence come to light

## Civility

*Civility is the ethical requirement to engage others with respect*. It implies “listening to the other person with a willingness to learn and to let one’s mind be changed” (Wolterstorff 1997, 112). It may also require asking for forgiveness if one showed lack of civility. In its broadest sense, civility furnishes an ethic for dialoguing across differences. Savulescu (2024, 38) describes global bioethics dialogue as involving “engaging in rational argument, often with someone with a radically different and challenging position. It is about the search for knowledge, not the presupposition of it.”

Bretherton (2009) usefully distinguishes three normative models for dialoguing across radical difference. First, *translation*, a normative model associated closely with Rawls (1971), holds that engaging with others ideally proceeds from behind a veil of ignorance, where people set aside “inessential” aspects of their identities and offer reasons that are compelling irrespective of any specific doctrines people hold. Exemplifying a translation model, Beauchamp and Childress characterize their four principles—autonomy, beneficence, nonmaleficence, and justice (hereafter principlism)—as “universal norms shared by all persons committed to morality” (Beauchamp and Childress 2019, 3). They argue that the four principles are “broad, abstract, and content-thin,” contrasting them with norms of particular moralities, which are “concrete, nonuniversal, and content rich” and reflect sociocultural moralities, religious traditions, and professional moralities (Beauchamp and Childress 2022, 165). Also exemplifying a translation approach, Crisp (2023) defends a “demoralizing” method whereby moral preferences are translated to remove moral terminology and focus just on satisfying preferences. This approach, Crisp maintains, is “less likely to be misled by emotion” (Crisp 2023, 6).

A translation model requires people to translate their moral concerns into a putatively shared language that all can accept, irrespective of their particular attachments or faith commitments. Yet, a challenge to translation views is that from a pluriversal or global standpoint, neither the four principles nor preference satisfaction may represent a neutral meeting ground for diverse moral traditions. For example, Fan, a Confucian bioethicist, dubs the four principles, “an abridged version of Modern Western liberal ethical norms” (Fan 2024, 355).

A second model for engaging across difference is a *conversation model.* It holds that people participating in ethics debates ought to express their ideas using their own vocabulary: “public discourse, if it is to be genuinely plural, cannot prescribe translation” (Biggar 2009, 170). A conversation model envisions people sharing their views in as much detail as they see fit and asking others to “try to make sense of each other’s perspectives,” while also being willing to “expose their own commitments to the possibility of criticism” (Bretherton 2009, 92). This approach is associated most closely with philosophers such as MacIntyre (1981) and Stout (2004). A challenge with a conversation model is that the moral traditions it seeks to engage operate amid power inequalities that centre some groups while sidelining others. The views that “win out” do not necessarily reflect the best or best justified claims, but rather claims that align with a prevailing hegemony (Bretherton 2009). A further challenge is that a conversation model might reach an impasse if people holding competing views become focused on winning over an opponent, rather than listening to understand.

A third model for engaging across difference, *hospitality*, provides the most compelling account. To be “hospitable” is literally to engage by “offering or affording welcome and entertainment to strangers” or being “disposed to receive or welcome kindly; open and generous in mind or disposition” (Oxford University Press 2023a, b). A hospitality model prescribes a substantive code for how people ought to conduct themselves in the public bioethics square. Like translation and conversation, it insists on respectful relations, yet goes further, demanding engaging “with the concrete reality of others rather than some abstracted homogenized, or idealized vision of them” (Bretherton 2009, 105). It does not seek a “winning” view but instead seeks to enrich a conversation by adding threads i.e., inviting others in.

## Change From Within

*Change from within is the ethical commitment to resolve conflict and reduce harm by working with those involved in conflict and experiencing harm*. Even when harms occur, and practices and policies require reform, it is often more effective, as well as ethically preferable, to identify and support drivers of change from within. In cases such as Tsux’iit/Luna, where two systems of law collide, the laws of both must be respected by seeking a compromise each side can agree to accept.

To further illustrate, consider the maltreatment of people, mostly women and girls, accused of witchcraft across Ghana’s Northern sector and in several other African countries. Roxburgh (2016, 894) depicts witchcraft not as a superstition but as “much more comprehensive, relating to human actions, intentions and experiences of the world. Thus, witchcraft is a reality, part of the world that actually exists, is experienced and though not seen, is evidenced in its outcomes.” Despite a 2023 Ghanian law criminalizing the declaration, accusation, naming, or labelling of another person as a witch (Ghana, Parliament 2023), belief in witches persists, deeply rooted in cultural practices ranging from popular music, socialization, and proverbs to patriarchal norms that sanction gynophobia and misogyny (Adinkrah 2015; Ghana Psychological Association 2020; ActionAid UK 2012; Amenga-Etego 2020). People accused of witchery are evicted from communities and face significant risk of physical assault, violence, torture, and murder (Roxburgh 2016; Duodu 2020; Whitaker 2020). In Ghana, individuals branded witches seek solace in witch-camps, where the chief protects them and provides bare necessities in exchange for work on his farms. ActionAid U.K. describes witch-camps as “effectively women’s prisons where inmates have been given no trial, have no right of appeal but have received a life sentence” (ActionAid UK 2012); at the same time, the group opposes disbanding camps, which would leave people accused of witchcraft vulnerable to harm.

Witchcraft raises clear concerns for bioethicists when individuals refuse medical care because they believe their ailments are due to witchery. Beyond this, physical and psychological violence against women and girls falls within the purview of bioethics because it undermines people’s health and lives. A pluriversal approach condemns maltreatment of people branded witches. It addresses the violence directed at them by first, listening to the accused to ensure interventions aimed at improving their plight do not unintentionally worsen it. Second, a pluriversal approach highlights solutions that emerge from understanding local epistemologies, practices, and ways of thinking. For instance, it engages with trusted authorities like churches that can help by absolving accused witches through exorcizing them of perceived powers. Nongovernmental organizations, such as go Home Project and HelpAge Ghana, also work with accused women and girls and their villages to bring about reconciliation and make peace between and within families, often by persuading the chief to accept the return of a witch, which signals that others in the community should too. Used in this way, belief in witchcraft can be a force channelled to “heal and protect” vulnerable people (Roxburgh 2016, 904–905). A pluriversal approach does not see its main mission as eradicating belief in witchcraft. Instead, the goal of a pluriversal approach is to navigate amid radically different ways of being, knowing, and acting to prevent harm and to support the coexistence of many worlds. Rather than imposing solutions “from the outside,” a pluriversal method supports individuals’ ability to determine for themselves the best way forward as well as which beliefs to hold on to or reject (Mabefam 2023).

## Justice

*Justice involves giving each their due and taking steps to ensure that each person feels they are given their due.* Because people are not only rational beings, but also creatures that emote and desire, justice strives to avoid insult, heal rifts, and foster a sense of belonging. Woodruff describes justice as “the virtue that sustains community” a kind of glue that prevents communities from fracturing (Woodruff 2014, 142). The conditions necessary for justice in this robust sense are twofold: a *dueness condition* requiring that each individual who wishes to contribute to bioethics has a fair chance to give testimony and have it received as prima facie credible and a *subjective condition* requiring each contributor to bioethics perceives and feels that the dueness condition was met for them. Both conditions are essential to harmony and community—dueness without a subjective sense of receiving one’s due can undermine community, while feeling recognized when one is not creates a false sense of community that is vulnerable to being exposed and undermined.

Woodruff astutely notes that the subjective condition is not based on procedures but on showing genuine responsiveness to others:

However well people are educated, they are subject to … the nonrational passions that arise from people’s love of honor and fear of shame … There is no simple algorithm for sustaining justice … [since] each human being is different from every other, and each makes a unique contribution to the whole. (Woodruf 2014, 145)

Contemporary debates about justice often overlook the subjective condition of justice to focus on procedural fairness. Yet the subjective condition matters too because people *deserve* to *feel heard and taken seriously*.

## Non-domination

*Non-domination prohibits others’ arbitrary and controlling influence.* It supports individuals and communities determining for themselves what bioethics concerns are salient for them, and what methods should be used in their resolution. Dworkin renders nondomination as a dimension of dignity and regards each person as having a special right and responsibility to exercise their own judgment about how to lead their life (Dworkin 2008a, b, 10).

Both avowed atheists and devout believers can flout the ethical constraint of non-domination. The moral violation does not arise from holding any particular worldview but from a mode of engaging (or not engaging) with those who hold different worldviews. As Biggar (2009, 161) puts it, “subscribers to world-views of all kinds … sometimes prefer to domineer than to reason together.” Biggar suspects that what fuels resistance to religion has less to do with religiously-based metaphysics than with anxiety about religion becoming “authoritarian, intolerant, divisive, bloody—and intolerable” such that “the basic good of civil peace demands that it be banned” (Biggar 2009, 151). The ethical constraint of non-domination condemns religious domination but also rejects dogmatic intolerance of religion. In both instances, the moral error is the same: dominating a bioethics conversation by silencing an opponent’s testimony. Bioethics, especially as it is practiced across a globally diverse landscape, must insist on discussions that are open to people holding radically different ways of being, knowing, and acting.

## Tolerance

*Toleration requires avoiding judging others with undue severity.* It involves displaying “freedom from bigotry” and “forbearance” (Oxford University Press 2024). Scanlon describes tolerance within a shared association as a normative requirement to recognize “common membership [with others] that is deeper than … conflict, a recognition of others as just as entitled as we are to contribute” (Scanlon 2003, 192).By contrast, the intolerant.

… claim a special place for their own values and way of life. Those who live in a different way … are, in their view, not full members of their society, and the intolerant claim the right to suppress these other ways of living. (Scanlon 2003, 192)

In a tolerant society, each party is “equally entitled to be taken into account in defining what our society is and equally entitled to participate in determining what it will become in the future” (Scanlon 2003, 190).

Across a pluriverse, toleration carries risk. It can lead to a future bioethics that is not what I, or my group, wants. Scanlon, apparently an atheist, puts the point bluntly:

I am content to leave others to the religious practices of their choice provided that they leave me free to enjoy none. But I would be very unhappy if this leads in time to my society becoming one in which almost everyone is, in one way or another, deeply religious … What I fear is not merely the legal enforcement of religion but its social predominance. (Scanlon 2003, 191–192)

Ultimately, what drives a tolerant bioethics community is a commitment to humility, i.e., to a sense that one does not know everything or possess privileged knowledge of normative truth. Tolerant people are humble in the sense that they put forth their views non-dogmatically and are open to revising them should new evidence or arguments come to light.

Together, these five ethical constraints serve as a bulwark against absolutism and zeal. They apply equally to religious and secular people, placing differences between them within a framework of mutual respect.

## A Pluriversal Approach to Religion

This section extends a pluriversal approach to religion and considers an opposing viewpoint.

## Arguments for a Pluriversal Approach to Religion

Three arguments lend support to applying a pluriversal approach to religious contriutions to bioethics. The first argument appeals to epistemic justice, the requirement to be fair and inclusive when assigning credibility to beliefs. Being epistemically just requires regarding other bioethicists as epistemic peers, i.e., “persons who are, in the matter in question, equally rational, possessed of the same relevant evidence, and equally conscientious in assessing that evidence” (Audi 2011, 117). Within a bioethics community, each person treats others’ normative views as *prima facie* equal to their own. This does not imply endorsing another’s views on reflection, but rather, engaging earnestly. The proposal to exclude religion disregards this requirement, positioning secular bioethicists as the final arbiters of what “bioethics” is.

A second argument appeals to standpoint epistemology. Standpoint epistemologists hold that knowledge is always socially produced and reflects the social position of those producing it. They tell us that people occupying privileged social positions often enjoy a monopoly on knowledge production and their claims are often considered universal, while those occupying marginalized social positions often receive less credibility and their knowledge claims are often considered applicable only within confined spaces. To illustrate, consider again Beauchamp and Childress’s principlism. As noted, Beauchamp and Childress maintain that their four principles—autonomy, beneficence, nonmaleficence, and justice—are content-thin, universal, and reflect a common morality. Standpoint epistemology destabilizes each claim. Rather than being content-thin and reflecting a universal morality, principlism omits religious standpoints and the moral perspectives associated with non-Western traditions. When secular Western sources dominate bioethics, they can unwittingly proliferate inequalities in knowledge production and sideline or erase standpoints other than their own. Avoiding the charge that “global bioethics” is nothing but the spread of Western ideas, i.e., “attempts by the dominant Western framework to morally colonize,” requires rejecting exclusionary value frameworks and building more pluriversal ones (Widdows 2007, 306). In contrast to universalizing frameworks, pluriversal frameworks incorporate a multiplicity of standpoints. For example, a pluriversal framework from Ghana might incorporate elements of Christianity, Islam, and African traditional religions and feature values such as accepting fate, deferring to authority, and fostering communitarian bonds (Atuire et al. 2020). While a pluriversal approach does not forbid holding *universal* principles it does proscribe *universalizing* them in ways that are uncivil, disrespectful, unjustly dominating, or intolerant of others.

A final argument holds that excluding religious contributions to bioethics hinders the field from gaining the global traction required to address bioethical problems that are increasingly global in scope. A claim to be “global” in one’s approach is suspect if it excludes religious sources of moral beliefs because most people around the world identify as religious. A 2017 Gallup International poll reported 62 per cent of the global population define themselves as religious, 74 per cent believe we have a soul, 71 per cent believe in God, 56 per cent believe in heaven, 54 per cent in life after death, and 49 per cent in hell (Gallup International 2017). It might be argued that although most of the global population is religious, most governments are not religiously affiliated. Perhaps, excluding religion remains viable when debating bioethics in the public square, e.g., discussing laws and public policies. Yet this response takes us only so far. In 2017, over eighty countries favoured a specific religion, either by endorsing it in their constitution or basic laws, or by providing affordances such as preferred legal or financial treatment (Pew Research Center 2017). Twenty-seven countries, mostly in the Middle East and North Africa (MENA) region, officially endorsed religion and thirteen, including nine European nations, endorsed Christianity or a Christian denomination. Leaving religious approaches out of global bioethics is objectionable because it silences the views of people living in the eighty countries where governments officially endorse religion and religion shapes law and public policy.

Even in secular societies where governments are prohibited from endorsing religion, and where citizens are putatively free to practice any religion, many people report religious belief or identify as religious. For example, the American Constitution does not refer to God or the divine, but in 2022, 81 per cent of Americans reported belief in God (Jones 2022). In 2020, half said the Bible should influence laws, including 28 per cent who favoured the Bible over the will of the people (Lipka 2020). The religiosity of American citizens is reflected at the state level with God or the divine referenced in all fifty U.S. state constitutions (Sandstrom 2017). The problem with excluding religious contributions to bioethics in secular societies like the United States is that it risks alienating most Americans, who turn to religious ideas to guide moral choices. For clinical bioethicists especially, engaging with religious ideas can be essential to helping patients, families, and clinicians wrestle with difficult medical decisions.

It is also worth noting that in many parts of the world, people do not draw bright lines between “secular” and “religious.” For example, adherents of African traditional religions do not regard their religious belief as something that can be “extracted from public life and quarantined in its own sphere,” because it suffuses virtually every aspect of daily life, from “birthing and death, marriage, family dynamics, diet, dress and grooming,” to “health care (including mental health), the spending and saving of money, interactions with one’s friends and neighbors, and … governance” (Olupona 2014, 2).

## An Opposing View

An opposing view defends keeping religion out of bioethics or at least cordoning it off into a separate “sphere” comprised of like-minded religiously inclined bioethicists. Schüklenk, Editor in Chief of both *Bioethics* and *Developing World Bioethics*, announced in 2018 that the latter journal would.

… significantly limit exclusively religious contributions …, precisely because they do not actually contribute to the conversations and dialogues that continue apace in our field. We will continue to publish such content, but for it to pass muster its arguments will have to have broader significance, beyond the followers of a particular religion. (Schüklenk 2018, 207).

Schüklenk’s defended this decision on the ground that.

Religion-based arguments are, by definition, arguments that do not fall into the category of public-reason based arguments. They rely on premises involving the existence of unobservable supernatural powers giving us direction in terms of how we must live our lives. Typically, their guidance is provided in religious documents the content of which is credited to said unobservable powers. (Schüklenk 2018, 207).

While admitting that this description of the world’s major monotheistic religions (Judaism, Christianity, and Islam) is simplified, Schüklenk insisted, “its key elements are pretty much like this” (Schüklenk 2018, 207).

Nor is Schüklenk alone in taking this stance. Cahill (1990, 11) has stated that religious approaches,

… will be appropriate and effective to the extent that they can be articulated in terms with a broad if not universal appeal. In other words, faith language that offers a particular tradition’s beliefs about God as the sole warrant for moral consideration will convince only members of that tradition.

Previously, Murphy published a Target article in *The American Journal of Bioethics* declaring that bioethics not only must have nothing to do with religion but is incompatible with it, “since moral theory intentionally employs only goals, methods, and evidence that prescind from theology” (Murphy 2012, 3). Murphy endorses “irreligious bioethics,” defined as “a lack of religious belief or being at variance with religious principles …,” including “disregard for religion or even a degree of hostility”(2012, 3). According to Murphy, “everyone [in bioethics] can benefit from irreligion” (2012, 3). Among the benefits Murphy touts are safeguarding the field from “ideological excesses;” furnishing “a detached vantage point from which to judge the value of religion;” and repudiating “any alleged transcendent reality—such as a world peopled by divinity, angels, demons, and human souls—as somehow relevant to the decisions to be made in biomedicine” (Murphy 2012, 3, 5, 6).

## Replies to the Opposing View

The problem with excluding religious contributions has been suggested already. It violates epistemic justice by deflating the credibility of religious testimony; cedes knowledge production to a relatively small group of people; and neglects stakeholders across the globe who are directly impacted by global bioethics problems like climate change, immigration, and antimicrobial resistance and who draw on their religious faith to craft a moral response to bioethics challenges. In addition to these objections, the practical implications of systematically excluding religious contributions would be unacceptable. To illustrate, consider what it might mean to exclude religious contributions from bioethics publishing, conferencing, and training programmes.

*(i) Bioethics publishing.* Consider first excluding religion from journal publishing. This might take the form of general-purpose bioethics journals excluding religious contributions, effectively cordoning religious contributions off into separate academic spaces. For example, religious contributions might be prohibited in *Developing World Bioethics* or *The American Journal of Bioethics* but permitted in *The Linacre Quarterly* (the official journal of the Catholic Medical Association); *Christian Bioethics* (an interdenominational journal exploring Christian faith); *or The Journal of Islamic Ethics*. Even without an official journal policy excluding religion, exclusion may occur if journal editors share Schüklenk’s views, or if peer reviewers do. It is difficult to gauge the extent to which this happens. What we do know is that relatively few bioethics journals explicitly *welcome* religious submissions. Instead, many bioethics journals seem to fall in the middle—neither explicitly inviting nor explicitly excluding religious submissions, and occasionally publishing them. For example, the *Journal of Bioethical Inquiry,* an official partner journal of the American Society of Bioethics and Humanities (ASBH), neither invites nor excludes religious submissions. The journal published a 2022 paper on balancing religious tolerance and patient care in the age of COVID-19 (Lederman and Halberthal 2022); a 2023 paper on Islamic perspectives on mitochondrial replacement therapy (Ibrahim et al. 2023); and a 2024 paper on Jewish perspectives on vaccinating inmates against COVID-19 (Rashi 2024). These and other examples (Gastmans et al. 2024; Ibrahim and Harun 2024; Komesaroff 2024; Rashi 2024; Ghaly 2014) suggest openness to religious contributions.

The difference between excluding, accepting without inviting, and explicitly inviting is nontrivial. *Excluding* is objectionable, because it allows secular bioethics to monopolize the field. *Accepting without inviting* seems better; however, it leaves intact the status quo, which is problematic if the status quo is unfriendly to religion. *Explicitly inviting* carries advantages, e.g., challenging the field’s starting assumptions and bridging differences between diverse worldviews. Explicitly inviting also prevents secularism from becoming oppressive. A deliberate commitment to being inclusive might be called for to overcome the tendency of dominant Western models, like principlism, to universalize secular principles and methods.

*(ii) Bioethics conferencing*. Suppose next, that organizers of international bioethics conferences excluded religious contributions. While we are not aware of conference organizers explicitly excluding religious submissions, objections have been raised to the selection of religious themes. For example, when the Qatari host of the 2024 World Congress of Bioethics, (WCB) proposed “Religion, Culture, and Global Bioethics” as the conference theme, and the Board of Directors of the International Association of Bioethics (IAB) accepted it, the decision met resistance (Jecker et al. 2023, 20). The IAB defended its decision on the ground that well-regarded professional bioethics groups make space for religious perspectives (IAB 2024). For example, the United Nations Educational, Scientific and Cultural Organization’s (UNESCO’s) Bioethics Chair sponsored a major initiative on “Bioethics, Multiculturalism and Religion,” and has held annual international conferences and workshops since 2009 addressing this topic (UNESCO n.d.); the European Society for Philosophy of Medicine and Healthcare (ESPMH) has included religion in its conferencing, selecting, “The Human Condition In Between Medicine, Arts and the Humanities” for its 2018 theme and including in its Call for Abstracts, “Religious/Theological Studies” (ESPMH 2018); and the ASBH has affinity groups with a religious focus, such as Bioethics and Christian Theology; Islamic Ethics; Jewish Bioethics; and Religion, Spirituality and Bioethics (ASBH n.d.).

In further defence of a religious theme for WCB 2024, IAB (2024) argued that religion shapes the belief of eight in ten people worldwide and informs health law and policy in many countries. Like many MENA countries, Qatar’s official state religion is Islam. Its constitution identifies Sharia law as “the main source of its legislations” (State of Qatar, 2004). In 2021, 12 per cent of Qatar’s population of 2.5 million were citizens, mostly Sunni Muslim; the remaining 88 per cent were noncitizens, mostly Shi’a Muslim (U.S. Department of State 2022). Bioethics arguments in MENA societies are apt to be more compelling to people in those societies and contribute more to shaping their public debate and policy if they employ partly religious, rather than purely secular, ideas. In majority religious societies like Qatar, consensus emerges through religious reasons being advanced without filters. Salam gives the example of Tunisia, where there is a law banning polygamy based on a particular interpretation of the Quran, making the point that, “the state justifies a law in terms of a religious argument based on reasons that appeal to the majority of citizens” (Salem 2019, 13). Like Salem, we support “an inclusive notion of public reason” (Salem 2019, 19) that regards religious reasons, in some circumstances, as part of the justification of public policies.

*(iii) Bioethics training programmes*. Finally, consider the implications of barring religious contributions from international training programmes. Two common models of international training are bringing students from low- and middle-income countries to high-income Western countries to learn about bioethics and developing programmes in trainees’ home countries. Both approaches tend to involve the export of Western texts and methods to countries outside the West. The first model includes trainees traveling to the United States via the Fogarty International Center of the National Institutes of Health; to Europe, via the Erasmus Mundus Master’s programme in bioethics; and to the United Kingdom via the Wellcome Trust. De Vries and Rott (2011) conducted interviews with twenty-one trainees at a European-based bioethics programme exploring the programme’s impact on trainees. Three reverberating themes emerged: materials used rendered knowledge from home countries invisible; methods taught were nearly always principlism, which was frequently at odds with methods and values prevalent in trainees’ countries; and trainees encountered resistance to requests to add non-Western sources to training materials. These findings defy claims that “secular” bioethics is content-thin or universally shared. They suggest instead an ongoing tension between diverse standpoints; more broadly, they convey that “both secular and religious bioethicists must not assume that the texts they hold dear apply universally or are univocal” (Duivenbode and Padela 2019, 2).

Establishing training programmes in low- and middle-income countries where trainees reside raises many of the same issues. For example, Jafarey and Mozam set up a Center of Biomedical Ethics and Culture at Aga Khan University Medical School in Karachi, Pakistan and reported a disconnect between the materials taught and the religious and cultural orientations of trainees (Jafarey and Mozam 2010). Additional concerns were the psychologically jarring aspect participants reported when required to pivot between secular-oriented bioethics training and religiously oriented clinical settings, often in the same day. Eventually, the remedy was changing the training model to resonate better with Pakistani society. For example, under the revised training model, trainees were invited to debate posthumous harvesting of sperm at a wife’s request by considering Muslim values that emphasize a strong family system and associated difficulties of being a single parent or fatherless child in Muslim society. Trainees discussed how Muslim values should change in response to women’s changing roles.

These examples attest that too often, international bioethics training has become “a process of integrating people around the world into a single society,” emphasizing “Americanization, Westernization,” or the ‘the power of transnational capitalism to distribute its cultural goods around the world’ (Bhakumi 2022, 65). According to Bhakumi, global bioethics education should instead travel elsewhere to uncover “the ethos of different local contexts to find the global in it” (Bhakumi 2022, 71). In many societies outside the West, values other than the four principles may play central roles: in Buddhism, a central concept is compassion; in Muslim societies, “first your neighbor…not first yourself”; in India, *Vasudhaiva kumtumbakam* (universal family) describes an interconnected, interdependent world and champions unity, compassion, and understanding (De Vries and Rott 2011, 9).

A secular bioethicist might counter that it is never acceptable for bioethical arguments to include premises referencing “unobservable supernatural powers” non-believers reject. In reply, boxing all religious people into this characterization is misguided. For example, some religious people draw on natural theology, which makes no recourse to revealed truths and specifically avoids appeals to “non-natural” facts, such as mystical experiences or supernatural beings; instead, natural theology adheres to “the same standards of rational investigation as other philosophical and scientific enterprises and is subject to the same methods of evaluation and critique” (Chignell and Pereboom 2020). Shi’ite Muslims and Roman Catholic theologians are among those who follow a natural theology approach, as do many classical and contemporary Western philosophers who put forth arguments for the existence of God, such as Plato, Anselm, Aquinas, Augustine, Descartes, Leibniz, Kant, Hume, and more recently, Adams, Plantinga, and van Inwagen (Chignell and Pereboom 2020). Within bioethics, notable scholars of natural theology include Ramsey (1993 [1950]) and McCormick (1989).

What about instances like witchcraft (discussed in Section I), where people manifest ways of being, knowing, and acting that depart radically from the analytic and empirical methods more familiar to mainstream bioethics? A pluriversal response condemns violence and abuse of people accused of witchcraft. It responds to witchcraft by seeking to instigate change from within that supports vulnerable people. Committing to change from within does not imply accepting religious way of knowing, nor does it imply belief in witches. Instead, a pluriversal method sets as a *normative* requirement supporting a plurality of worlds, provided they avoid harming people or destroying other worlds.

With these ethical constraints in place, efforts to welcome religious contributions in bioethics publishing, conferencing, and training help establish bioethics as a field friendly to difference, including religious difference.

## Conclusion

Our chief aim in this paper was setting forth and defending a pluriversal approach to religion in the context of an increasingly global bioethics. A pluriversal approach instructs bioethicists to navigate all worlds, including religious worlds, with civility. It demands working from within, being just, not dominating, and showing tolerance. All bioethicists, especially those concerned with ethical problems with global reach, should commit to a bioethics pluriverse and take steps to realize it.

## Data Availability

Not applicable.

## Footnotes

1 “Destroying other worlds” means literally eliminating other ways of knowing, being, and acting. Civilizing colonialism, for example, saw colonial rule as a civilizing mission, in which the West sought to pass down European value systems and educate barbarian peoples. In the process, it eliminated indigenous people’s ways of knowing, being, and acting. See Ypi, L. 2013. What’s wrong with Colonialism. *Philosophy and Public Affairs* 41(2): 158–191.

## Declarations

Ethics Approval

## References

[CR1] ActionAid U.K. 2012. Condemned without trial: Women and witchcraft in Ghana. https://www.actionaid.org.uk/sites/default/files/publications/condemned_without_trial_women_and_witchcraft_in_ghana_report_september_2012.pdf. Accessed November 24, 2024.

[CR2] Adinkrah, M. 2015. *Witchcraft, witches, and violence in Ghana*. Brooklyn: Berghahn Books, Inc.

[CR3] Amenga-Etego, R.M. 2020. Witchcraft and modernity in Ghana: A people’s approach to witchcraft and fear. In *Witchcraft Accusations in Global Perspective*, edited by K. Werner and G. Priemer, 117–136. Hamburg: Missionshilfe Verlag.

[CR4] American Society for Bioethics and Humanities (ASBH). No date. Affinity Groups. https://asbh.org/membership/affinity. Accessed November 24, 2024.

[CR5] Atuire, C.A., C. Kong, and M. Dunn. 2020. Articulating the sources for an African normative framework of healthcare: Ghana as a case study. *Developing World Bioethics* 20(4): 216–227. 32511832 10.1111/dewb.12265

[CR6] Audi, R. 2011. *Democratic authority and the separation of church and state*. New York: Oxford University Press.

[CR7] Audi, R., and W.R. Smith. 2023. Religious pluralism and the ethics of healthcare. *Bioethics* 37: 42–51. 36490383 10.1111/bioe.13113 PMC10841336

[CR8] Beauchamp, T., and J. Childress. 2022. Common morality principles in biomedical ethics: Responses to critics. *Cambridge Quarterly of Healthcare Ethics* 31(2): 164–176. 34511156 10.1017/S0963180121000566

[CR9] ———. 2019. *Principles of Biomedical Ethics*, 8th ed*.* New York: Oxford University Press.

[CR10] Bhakumi, H. 2022. Glocalization of bioethics. *Global Bioethics* 33(1): 65–77. 10.1080/11287462.2022.2052603 PMC8942517 35340843

[CR11] Biggar, N. 2015. Why religion deserves a place in secular medicine. *Journal of Medical Ethics* 41: 229–233. 24763220 10.1136/medethics-2013-101776

[CR12] ———. 2009. Not translation, but conversation: Theology in public debate about euthanasia. In *Religious Voices in Public Places*, edited by N. Biggar and L. Hogan, 151–193. New York: Oxford University Press.

[CR13] Blaser, M. 2013. Ontological conflict and the stories of people in spite of Europe: Toward a conversation on political ontology. *Current Anthropology* 54(5): 547–568.

[CR14] Bretherton, L. 2009. Translation, conversation, or hospitality? Approaches to theological reasons in public deliberation. In *Religious Voices in Public Places*, edited by N. Biggar and L. Hogan, 85–109. New York: Oxford University Press.

[CR15] Cahill, L. 1990. Can theology have a role in “public” bioethics discourse? *Hastings Center Report* 20(4): 10–14. 2211074

[CR16] Callahan, D. 1999. The social sciences and the task of bioethics. *Daedalus* 128(4): 275–294. 11645879

[CR17] Camosy, C.C. 2012. The role of normative traditions in bioethics. *American Journal of Bioethics* 12(12): 13–15. 10.1080/15265161.2012.725349 23215920

[CR18] Chapman, A. 2012. In defense of the role of a religiously informed bioethics. *American Journal of Bioethics* 12(12): 26–28. 10.1080/15265161.2012.719275 23215927

[CR19] Chignell, A., and D. Pereboom. 2020. Natural theology and natural religion. In *Stanford Encyclopedia of Philosophy*, edited by E.N. Zalta, E.N. https://plato.stanford.edu/archives/fall2020/entries/natural-theology/. Accessed June 13, 2024.

[CR20] Claasen-Lütner, J.C. 2012. How religious ethics can be intelligible and compatible with bioethics. *American Journal of Bioethics* 12(12): 30–31. 10.1080/15265161.2012.719279 23215929

[CR21] Crane, J.K., and S.B. Putney. 2012. Exorcising doubts about religious bioethics. *American Journal of Bioethics* 12(12): 28–30. 10.1080/15265161.2012.719274 23215928

[CR22] Crisp, R. 2023. Religious preferences in healthcare: A welfarist approach. *Bioethics* 37: 5–11. 36383689 10.1111/bioe.13114 PMC10100007

[CR23] De Vries, R., and L. Rott. 2011. Bioethics as missionary work: The export of western ethics to developing countries. In *Bioethics Around the Globe*, edited by C. Myser C, 3–18. New York: Oxford University Press.

[CR24] Duivenbode, R., and A. Padela. 2019. Contextualizing the role of religion in the global bioethics discourse: A response to the new publication policy of *Developing World Bioethics*. *Developing World Bioethics* 19(4): 189–191. 31503370 10.1111/dewb.12242

[CR25] Duodu, S. 2020. 90-year-old woman accused of witchcraft lynched at Kafaba near Salaga. *Graphic Online*, July 24. https://www.graphic.com.gh/news/general-news/90-year-old-woman-accused-of-witchcraft-lynched-at-kafaba-near-salaga.html. Accessed November 24, 2024.

[CR26] Durante, C. 2012. Extending the hermeneutics of suspicion beyond irreligiosity. *American Journal of Bioethics* 12(12): 19–20. 10.1080/15265161.2012.719276 23215923

[CR27] Dworkin, R. 2008a. Common ground. In *Is Democracy Possible Here? Principles for a New Political Debate*, edited by R. Dworkin, 1–23. Princeton: Princeton University Press.

[CR28] ———. 2008b. Religion and dignity. In *Is Democracy Possible Here? Principles for a New Political Debate*, edited by R. Dworkin, 52–89. Princeton: Princeton University Press.

[CR29] Escobar, A. 2020. *Pluriversal politics. The real and the possible*. Durham: Duke University Press.

[CR30] European Society for Philosophy of Medicine and Healthcare (ESPMH). 2018. 32nd European Conference on Philosophy of Medicine and Health Care. https://www.espmh.org/wp-content/uploads/Call-for-abstracts-Lisbon-2018-docx.pdf. Accessed June 13, 2024.

[CR31] Fan, R. 2024. Principlism as global bioethics: A critical appraisal from a Confucian perspective. *Dao* 23: 353–3776.

[CR32] Fletcher, J.F. 1997*. Situation ethics: The new morality,* 2nd ed*.* Louisville, KY: Westminster John Knox Press.

[CR33] Gallup International. 2017. Religion prevails in the world. https://web.archive.org/web/20171114113506/http://www.wingia.com/web/files/news/370/file/370.pdf. Accessed June 13, 2024.

[CR34] Gastmans, C., E. Sinibaldi, R. Lerner, et al. 2024. Christian anthropology-based contributions to the ethics of socially assistive robots in care for older adults. *Bioethics.* ePub ahead of print, November 9. 10.1111/bioe.13322. 10.1111/bioe.13322 38857488

[CR35] Ghaly, M. 2014. Islamic bioethics: The inevitable interplay of “texts” and “contexts.” *Bioethics* 28(2): ii–v. 24383401 10.1111/bioe.12081

[CR36] Ghana Psychological Association. 2020. Let’s end witchcraft accusations. https://www.modernghana.com/news/1027491/lets-end-witchcraft-accusations.html. Accessed November 24, 2024.

[CR37] Ghana, Parliament. 2023. Amendment to the criminal offence (amendment) bill, 2022, memorandum. July 28. https://ir.parliament.gh/bitstream/handle/123456789/2385/Criminal%20Offence%20%28Amendment%29Bill%2C2022.pdf?sequence=1&isAllowed=y.

[CR38] Ibrahim, A.H., and M.S. Harun. 2024. Applying the concepts of benefit and harm in Malaysian bioethical discourse: Analysis of Malaysian Fatwa. *Journal of Bioethical Inquiry*. ePub ahead of print, November 24. 10.1007/s11673-024-10345-z. 10.1007/s11673-024-10345-z 38407763

[CR39] Ibrahim, A.H., N.N.A. Rahman, and S.M. Saifuddeen. 2023. Mitochondrial replacement therapy: An Islamic perspective. *Journal of Bioethical Inquiry* 20(3): 485–495. 37440155 10.1007/s11673-023-10279-y

[CR40] International Association of Bioethics. 2024. Frequently asked questions: Why did the 2024 IAB WCB select the theme, religion, culture, and global bioethics? https://iabioethics.org/2024-congress. Accessed June 13, 2024.

[CR41] Jafarey, A.M., and F. Mozam. 2010. “Indigenizing” bioethics: The first center for bioethics in Pakistan. *Cambridge Quarterly of Healthcare Ethics* 19(3): 353–362. 20507683 10.1017/S0963180110000137

[CR42] Jecker, N.S., V. Ravitsky, M. Ghaly, J.C. Belislé-Pipon, and C. Atuire. 2023. Proposed principles for international bioethics conferencing. *American Journal of Bioethics* 24(4): 13–28. 10.1080/15265161.2023.2232748 37549186

[CR43] Jones, D.G., and M. Whittaker. 2012. Reorienting bioethics by releasing it from any religious moorings. *American Journal of Bioethics* 12(12): 24–26. 10.1080/15265161.2012.719269 23215926

[CR44] Jones, J.M. 2022. Belief in God in U.S. dips to 81%, a new low. Gallup. https://news.gallup.com/poll/393737/belief-god-dips-new-low.aspx#:~:text=WASHINGTON%2C%20D.C.%20%2D%2D%20The%20vast,of%20Americans%20believed%20in%20God. Accessed June 13, 2024.

[CR45] Jonsen, A.R. 2003. *The birth of bioethics*. New York: Oxford University Press.

[CR46] Komesaroff, P.A. 2024. It is not too late for reconciliation between Israel and Palestine, even in the darkest hour. *Journal of Bioethical Inquiry*. ePub ahead of print, November 24. 10.1007/s11673-024-10347-x. 10.1007/s11673-024-10347-x PMC11052763 38517636

[CR47] Lederman, Z., and M. Halberthal. 2022. A close shave: Balancing religious tolerance and patient care in the age of COVID-19. *Journal of Bioethical Inquiry* 19(4): 625–633. 35852780 10.1007/s11673-022-10201-y

[CR48] Lipka, M. 2020. Half of Americans say bible should influence U.S. laws, including 28% who favor it over the will of the people. PEW Research Center. https://www.pewresearch.org/short-reads/2020/04/13/half-of-americans-say-bible-should-influence-u-s-laws-including-28-who-favor-it-over-the-will-of-the-people/. Accessed June 13, 2024.

[CR49] Mabefam M.G. 2023. *Witch camps and witchcraft discourses in Africa*. Lanham, MD: Rowman & Littlefield Publishing Group, Inc.

[CR50] MacIntyre, A. 1981. *After virtue: A study in moral theory*. London: Duckworth Books Group.

[CR52] McCormick, R. 1989. Theology and bioethics. *Hastings Center Report* 19(2): 5–10. 2649455

[CR53] Murphy, T.F. 2012. In defense of irreligious bioethics. *American Journal of Bioethics* 12(12): 3–10. 10.1080/15265161.2012.719262 23215918

[CR54] Olupona, J.K. 2014. *African religions: A very short introduction*. New York: Oxford University Press.

[CR55] Oxford University Press, 2023a. About *Christian bioethics*. https://academic.oup.com/cb/pages/About. Accessed 3 Dec 2024.

[CR56] ———. 2024. Tolerance, n. In *Oxford English Dictionary*. New York: Oxford University Press. 10.1093/OED/9975949183.

[CR57] ———. 2023b. Hospitable, adj. In *Oxford English Dictionary*. New York: Oxford University Press. 10.1093/OED/1047462463.

[CR58] Pew Research Center. 2017. Many countries favor specific religions, officially or unofficially. Pew Research Center, October 3. https://www.pewresearch.org/religion/2017/10/03/many-countries-favor-specific-religions-officially-or-unofficially/. Accessed June 13, 2024.

[CR59] Ramsey, P. 1993 (1950). *Basic Christian Ethics*. Louisville, KY: Westminster Publishing, John Knox Press.

[CR60] Rashi, T. 2024. Jewish ethics of inmate vaccines against COVID-19. *Journal of Bioethical Inquiry* 21: 57–66. 10.1007/s11673-023-10331-x. 38427178 10.1007/s11673-023-10331-x PMC11052822

[CR61] Rawls, J. 1971. *A theory of justice*. Cambridge: Harvard University Press.

[CR63] Roxburgh, S. 2016. Witchcraft and violence in Ghana. *Cahiers d'études Africaines* 56(224): 891–914.

[CR64] Salem, D. 2019. Public reason: A stranger in non-liberal and religious societies? *Philosophy and Social Criticism* 45(1): 3–26.

[CR65] Sandstrom, A. 2017. God or the divine is referenced in every state constitution. PEW Research Center, August 17. https://www.pewresearch.org/short-reads/2017/08/17/god-or-the-divine-is-referenced-in-every-state-constitution/#:~:text=In%20fact%2C%20God%20or%20the,“God”%20at%20least%20once. Accessed June 13, 2024.

[CR66] Savulescu, J. 2024. Two models of bioethics. *American Journal of Bioethics* 24(4): 37–38. 10.1080/15265161.2024.2312765 38529974

[CR67] ———. 2023. Autonomy, well-being, justice, professional responsibility and personal values: A commentary on Roger Crisp, “Religious preference in health care: A welfarist approach.” *Bioethics* 37: 12–14. 36440968 10.1111/bioe.13119

[CR68] Scanlon, T. 2003. *The difficulty of tolerance*. Cambridge: Cambridge University Press.

[CR69] Schüklenk, U. 2018. On the role of religion in articles this journal seeks to publish. *Developing World Bioethics* 18(3): 207. 30253055 10.1111/dewb.12210

[CR70] State of Qatar. 2004. The Constitution, Article 1, June 8. https://www.gco.gov.qa/wp-content/uploads/2016/09/GCO-Constitution-English.pdf. Accessed June 13, 2024.

[CR71] Stempsey, W.E. 2011. Religion and bioethics: Can we talk? *Journal of Bioethical Inquiry* 8(4): 339–350.

[CR72] Stout, J. 2004. *Democracy and tradition*. Princeton: Princeton University Press.

[CR73] ten Have, H. 2016. *Global bioethics: An introduction*. New York: Routledge, Taylor & Francis Group.

[CR74] U.S. Department of State, Office of International Religious Freedom. 2022. *The 2021 report on international religious freedom: Qatar*. Office of International Religious Freedom, June 2. https://www.state.gov/reports/2021-report-on-international-religious-freedom/qatar/. Accessed June 13, 2024.

[CR75] UNESCO Bioethics Chair. No date. Bioethics, multiculturalism and religion. https://www.unescobiochair.org/bioethics-multiculturalism-and-religion-3/. Accessed June 13, 2024.

[CR76] Whitaker, K. 2020. Ghana witch camps: Widows lives in exile. BBC News, September 1. https://www.bbc.com/news/magazine-19437130. Accessed 24 Nov 2024.

[CR77] Widdows, H. 2007. Is global ethics moral neo-colonialism? An investigation of the issue in the context of bioethics. *Bioethics* 21(6): 305–315. 17845454 10.1111/j.1467-8519.2007.00558.x

[CR78] Wolterstorff, N. 1997. The role of religion in decision and discussion of political issues. In *Religion in the Public Square: The Place of Religious Convictions in Political Debate*, edited by N. Wolterstorff and R. Audi, 67–120. Rowman & Littlefield Publishers.

[CR79] Woodruff, P. 2014. *The Ajax dilemma: Justice, fairness, and rewards*. New York: Oxford University Press.

